# Protection against Acute Hepatocellular Injury Afforded by Liver Fibrosis Is Independent of T Lymphocytes

**DOI:** 10.1371/journal.pone.0165360

**Published:** 2016-10-28

**Authors:** Benoit Lacoste, Valérie-Ann Raymond, Pascal Lapierre, Marc Bilodeau

**Affiliations:** 1 Laboratoire d’hépatologie cellulaire, Centre de Recherche du Centre Hospitalier de l'Université de Montréal (CRCHUM), Montréal, QC, Canada; 2 Département de Médecine, Université de Montréal, Montréal, QC, Canada; University of Navarra School of Medicine and Center for Applied Medical Research (CIMA), SPAIN

## Abstract

Collagen produced during the process of liver fibrosis can induce a hepatocellular protective response through ERK1 signalling. However, the influence of T cells and associated cytokine production on this protection is unknown. In addition, athymic mice are frequently used in hepatocellular carcinoma xenograft experiments but current methods limit our ability to study the impact of liver fibrosis in this setting due to high mortality. Therefore, a mouse model of liver fibrosis lacking T cells was developed using Foxn1 nu/nu mice and progressive oral administration of thioacetamide (TAA) [0.01–0.02%] in drinking water. Fibrosis developed over a period of 16 weeks (alpha-SMA positive area: 20.0 ± 2.2%, preCol1a1 mRNA expression: 11.7 ± 4.1 fold changes, hydroxyproline content: 1041.2 ± 77μg/g of liver) at levels comparable to that of BALB/c mice that received intraperitoneal TAA injections [200 μg/g of body weight (bw)] (alpha-SMA positive area: 20.9 ± 2.9%, preCol1a1 mRNA expression: 13.1 ± 2.3 fold changes, hydroxyproline content: 931.6 ± 14.8μg/g of liver). No mortality was observed. Athymic mice showed phosphorylation of ERK1/2 during fibrogenesis (control 0.03 ± 0.01 vs 16 weeks 0.22 ± 0.06AU; *P*<0.05). The fibrosis-induced hepatoprotection against cytotoxic agents, as assessed histologically and by serum AST levels, was not affected by the absence of circulating T cells (anti-Fas JO2 [0.5μg/g bw] for 6h (fibrotic 4665 ± 2596 vs non-fibrotic 13953 ± 2260 U/L; *P*<0.05), APAP [750 mg/kg bw] for 6 hours (fibrotic 292 ± 66 U/L vs non-fibrotic 4086 ± 2205; *P*<0.01) and CCl_4_ [0.5mL/Kg bw] for 24h (fibrotic 888 ± 268 vs non-fibrotic 15673 ± 2782 U/L; *P*<0.001)). In conclusion, liver fibrosis can be induced in athymic Foxn1 nu/nu mice without early mortality. Liver fibrosis leads to ERK1/2 phosphorylation. Finally, circulating T lymphocytes and associated cytokines are not involved in the hepatocellular protection afforded by liver fibrosis.

## Introduction

Liver fibrosis is characterized by the accumulation of extracellular matrix (ECM) produced by the inflammatory response caused by chronic liver injury [1–3]. This pathophysiological response is mainly observed in humans with chronic viral hepatitis, non-alcoholic steatohepatitis and alcohol abuse. Cirrhosis is the most severe stage of liver fibrosis and is strongly associated with the risk of developing hepatocellular carcinoma (HCC), a cancer associated with poor prognosis [4].

Liver fibrogenesis involves the activation of non-parenchymal cells together with coordinated secretion of cytokines [2, 5, 6]. Kupffer cells (KC) are tissue macrophages that become activated by lipopolysaccharides (LPS) and/or endotoxins through binding of pattern recognition receptors (PRRs), e.g., toll-like receptor 4 (TLR4). KC can also be activated by damage-associated-pattern-molecules (DAMPs) from intracellular constituents released by necrotic cells [7]. Once activated, KC secrete TGF-β1 and proinflammatory molecules, such as TNF-α, which then activate hepatic stellate cells (HSC) [1, 8, 9]. Activated HSC lose their vitamin A content, express alpha-smooth muscle actin (alpha-SMA) and become major producers of extracellular matrix (ECM) components [3, 10–12].

T lymphocytes can influence the development of fibrosis. CD4+ Th2 lymphocytes can secrete IL-13, IL-4 and IL-5, which have been shown to be fibrogenic [13, 14]. Pro-inflammatory CD4+ Th17 cells are also associated with several liver diseases such as alcoholic liver disease, viral hepatitis and non-alcoholic steatohepatitis [15].

Many experimental models have been developed to study liver fibrosis [16]. Thioacetamide (TAA), a known hepatotoxin, has been widely used in such context: the hallmarks of the fibrosis process induced by TAA are well characterized [17]. Susceptible mice strains, such as BALB/c, receive intraperitoneal (IP) injections of TAA. The toxic metabolite of TAA, TASO2, exerts its cytotoxic effect on hepatocytes which then leads to KC activation, cytokine production (such as TNF-α and TGF-β1) and the subsequent activation of HSC [18–21]. This ultimately leads to deposition of ECM compounds. When TAA injections are stopped, deactivation and apoptosis of HSC is observed followed by reversal of fibrosis [22–24].

Liver fibrosis has recently been shown to lead to an increased resistance to acute liver injury *in vivo* through ERK1/2 activation, which is now being considered a key marker of established fibrosis [25]. The influence of T lymphocytes and/or cytokines secreted by these cells on the hepatocellular protection afforded by liver fibrosis is currently unknown. Foxn1 nu/nu mice (nude mice) are athymic and therefore lack mature T-cells [26]. Immunodeficient mice are frequently used in hepatocellular carcinoma (HCC) xenograft models but evaluating the impact of fibrosis in these models is difficult since currently used methods to induce fibrosis (IP injections of TAA at 200 μg/g bw) lead to high mortality rates in these animals [27]. Therefore the aims of this study were 1) to develop a method for the induction of fibrosis in athymic mice without significant mortality; 2) asses and characterize the process of liver fibrosis induced in these mice compared to immunocompetent models of fibrosis and 3) evaluate the role, if any, of T lymphocytes on the protection afforded by liver fibrosis against acute hepatocellular injury [25].

Herein, we show that using progressive oral administration of low-dose TAA, liver fibrosis can be safely induced in Foxn1 nu/nu mice. The level of liver fibrosis is comparable to that obtained by TAA IP administration in BALB/c mice. Liver fibrosis induces the phosphorylation of ERK1/2 and confers hepatocellular protection to cytotoxic agents. Therefore, models based on Foxn1 nu/nu mice can be useful for the study of liver fibrosis and T cells are not involved in the hepatocellular protection afforded by liver fibrosis.

## Materials and Methods

### Animals

Male Foxn1 nu/nu and BALB/c mice (20 g) were purchased from Charles-River (Saint-Constant, Québec, Canada) and were fed *ad libidum* with normal chow in vented cages in the immunodeficient specific area of the animal welfare facilities at CRCHUM. Animals were monitored daily for their appearance, state of hydration, behavior and clinical signs. Humane endpoints were in place during the study. Endpoints included loss of 20% or more of body weight, aggressiveness associated with pain or uncontrollable pain, prolonged anorexia, prostration, dehydration and nervous disorder. Animals were sacrificed by exsanguination under anesthesia. All procedures were performed in accordance with Canadian Council on animal care and approved by the *Comité institutionnel de protection animale (CIPA) du CHUM (Canada)*.

### Chemicals

Fas JO2 antibody was purchased from BD Biosciences (Mississauga, Ontario, Canada). TRIZOL reagent was from Invitrogen (Burlington, Ontario, Canada). Quantitect reverse transcription kit and SYBRGreen kit were purchased from QIAGEN (Toronto, Ontario, Canada). Enhanced chemiluminescence (ECL) reactive was from Perkin-Elmer (Woodbridge, Ontario, Canada). Developer and fixator solution kits were from Kodak (Rochester, NY). Unless stated otherwise, all other products were from Sigma-Aldrich (Oakville, Ontario, Canada).

### Fibrogenesis

To induce fibrosis in Foxn1 nu/nu mouse, sterile filtered (0.2 μm) 2% TAA stock solution in spring water (low iron) was prepared and stored at room temperature. The TAA dilution [0.02%] was renewed every 2 weeks. Animals (1/cage) received the 0.02% TAA solution as regular drinking water; bottles were changed once every two weeks. To facilitate TAA intake and reduce mortality due to toxicity, mice received a solution of 0.01% TAA during the first 2 weeks, and then the dose was increased to 0.02% in their drinking water for 16 weeks. Animals were sacrificed at 4, 8, 10 and 16 weeks following administration of 0.02% TAA treatment (2-week 0.01% TAA period excluded). Two other groups of animal received normal drinking water for 2 or 3 weeks after the end of TAA administration to assess fibrosis regression. These animals were compared to a control group of Foxn1 nu/nu animals receiving normal drinking water for the entire treatment period. To induce fibrosis in BALB/c mouse, animals (2/cage) received, thrice a week, TAA IP injection [200 μg/g bw] as a 2% sterile filtered (0.2 μm) solution in isotonic saline buffer stored at room temperature. Control group animals received saline injection for the same period. Animals were sacrificed at 8, 10 and 12 weeks following the IP injection treatment. A group had a 2-week recovery after 12 weeks of injection to evaluate fibrosis regression.

### Acute liver injury

Following the 16-week fibrogenic TAA protocol, Foxn1 nu/nu mice were injected IP with the following cytotoxic agents: CCl_4_ [0.5 mL/kg bw] as a 20% solution in sterile mineral oil for 24 hours, Fas Jo2 antibody [0.5 μg/g bw] for 6 hours and APAP [750 mg/kg bw] for 6 hours.

### Histology

Formalin-fixed liver specimens were sliced, mounted on slides and stained (Pathology Department of CHUM-Saint-Luc Hospital). Masson’s trichrome staining was performed to assess the extent of fibrosis while hematoxylin-phloxin-safranin (HPS) staining was used for histological evaluation of liver injury. Pictures were taken using a Carl Zeiss Axioplan 2 (Göttingen, Germany) microscope at 100x magnification with Northern Eclipse 6.0 software (Empix Imaging, Missisauga, Ontario, Canada).

### Immunofluorescence

Slides of formalin-fixed and paraffin-embedded liver samples were dewaxed in xylene for 10 minutes. Slides were then rehydrated in successive baths of ethanol and water (100% for 6 minutes; 95% for 3 minutes, 70% for 3 minutes and water for 3 minutes). Slides were then blocked for 60 minutes in immunofluorescence buffer (IF buffer) that consists of PBS with 3% BSA and 0.05% Tween-20. Primary mouse anti-alpha-SMA antibody (Dako, Burlington, Ontario, Canada) was incubated overnight at a 1:100 dilution in IF buffer at 4°C. Slides were washed 3 times for 3 minutes and then incubated for 120 minutes with secondary FITC-labelled antibody (Dako, Burlington, Ontario, Canada) at a 1:20 dilution in IF buffer in the dark at room temperature. Slides were washed in PBS 0.05% Tween (PBST) and nuclei were stained with Hoechst 33258 [10 ng/mL] in PBS for 60 minutes in the dark. Slides were washed twice for 5 minutes in PBS and pictures obtained using Northern Eclipse 6.0 software (Empix Imaging, Mississauga, Ontario, Canada) were obtained by fluorescent microscopy (Carl Zeiss Axioplan 2, Göttingen, Germany). Image analysis was performed with ImageJ 1.46 software (NIH, USA) using 5 representative pictures at 100x magnification of each slide. Results were expressed as percentage of positive pixels for alpha-SMA of the total picture area.

### Hydroxyproline content

The hydroxyproline content was expressed as μg/g of liver as previously reported [28]. Briefly, 20 mg of frozen liver samples were hydrolysed in 6N HCl at 110°C in an autoclave at a pressure of 1.2 kgf/cm^2^ during 16 hours. After centrifugation at 2000 rpm at 4°C for 5 minutes, 2 mL of supernatant was transferred to another tube. Samples were neutralized (pH 7–8) using 8N KOH added with 1% phenolphthalein. Two mL of this solution was then stirred with 2 g of KCl and 1 mL of 0.5M borate buffer (pH 8.2) for 15 minutes at room temperature followed by another 15 minutes at 0°C. Chloramine T [0.2M] solution (1 mL) was added and stirred for 60 minutes at 0°C. The resulting solution was then mixed with 3.6M sodium thiosulfate (2 mL) and incubated for 30 minutes at 120°C. Toluene (3 mL) was added to the mixture for 20 minutes. Finally, Ehrlich’s solution (0.8 mL) was added to 2 mL of supernatant obtained after centrifugation at 2000 rpm at 4°C and left for 30 minutes at room temperature and stirred 5 times during this period. Absorbance of the lower phase was measured at 560 nm.

### Gene expression

Frozen liver samples (80–90 mg) were homogenized with a potter in 1 mL of TRIZOL reagent at 4°C in accordance with manufacturer’s recommendations. Two hundred μL of chloroform were added to the solution for RNA extraction, mixed thoroughly and centrifuged for 15 minutes at 14 000 g at 4°C. The aqueous phase was removed and incubated with 1:3 volume of isopropanol for 10 minutes at room temperature and then centrifuged for 10 minutes at 14 000 g at 4°C. The RNA pellets obtained were washed in 75% ethanol and re-suspended in sterile RNAse/DNAse-free water. According to the manufacturer’s recommendations of the Quantitect reverse transcription kit, 250 ng of each RNA sample was treated to remove genomic DNA and transcribed in DNA. Gene expression was analyzed following Quantitect SYBRGreen kit manufacturer’s guide with a RotorGene (Corbett Life Science, CA) Real-time PCR. For each gene tested, 35 cycles with a 59°C melting temperature were performed. Mouse preCol1a1 gene (F:5’-CATGTTCAGCTTTGTGGACCT-3’; R:5’-GCAGCTGACTTCAGGGATGT-3’) was evaluated using these reference genes: HPRT1 (F:5’-GCTTGCTGGTGAAAAGGACCTCTCGAAG-3’; R:5’-CCCTGAAGTACTCATTATAGTCAAGGGCAT-3’, Ppia (F:5’-CGCGTCTCCTTCGAGCTGTTTG-3’; R:5’-TGTAAAGTCACCACCCTGGCACAT) and H2afz (F:5’-ACAGCGCAGCCATCCTGGAGTA-3’; R:5’-TTCCCGATCAGCGATTTGTGGA-3’) [29]. Relative gene expression was measured using the Delta Delta CT method [30].

### Western blot analysis

Frozen liver samples (100 mg) were homogenized in 300 μL of complete RIPA buffer using a potter and dosed according to Bradford [31]. Protein extracts were prepared, subjected to a 12% SDS-polyacrylamide gel electrophoresis and then blotted as described [32]. Briefly, after a 1-hour block in 10% milk in PBST at room temperature, membranes were probed with the following antibodies: anti-ERK1/2, anti-phosphospecific ERK1/2 (both from Cell Signalling, Pickering, Ontario, Canada) and anti-actin kit from Calbiochem (Merck KGaA, Darmstadt, Germany) for 2 hours in PBST containing 1% milk at room temperature. Membranes were washed 3 times for 10 minutes in PBST and then incubated with a secondary antibody (HRP-conjugated anti-rabbit IgG or anti-mouse IgG both from BD Pharmingen, CA) or anti-mouse IgM from anti-actin kit for 1 hour in PBST containing 5% milk at room temperature. Finally, after 3 washes in PBST, membranes were incubated in presence of enhanced chemiluminescence (ECL) reactive. In a dark room, Hyblot CL films (Denville scientific Inc., Metuchen, NJ) were exposed to chemiluminescent membranes and then developed and fixed using Kodak solutions kits. Band densitometry was measured using ImageJ 1.46 software (NIH, USA).

### Aspartate transaminase (AST) and alanine transaminase (ALT) activities

Blood was obtained at sacrifice, left at room temperature for 30 minutes and then centrifuged for 5 minutes at 1400 rpm to isolate serum. Serum samples were then diluted 1:10 for testing. Quantitative determination of ALT and AST activities was performed using an automatic multianalyzer (Synchron LX System, Beckman Coulter, Mississauga, Ontario, Canada) by the Biochemistry Department of CHUM Saint-Luc Hospital

### Statistical analysis

All data represent the values of at least 4 experiments, each from different animals. Results are expressed as means ± SE. Differences among groups were analyzed using one- and two-way ANOVA for repeated measures or Student’s *t*-test. A *P-*value below 0.05 was considered significant.

## Results

### Induction of liver fibrosis in Foxn1 nu/nu mice using orally administered TAA

To induce liver fibrosis and reduce mortality due to toxicity, mice received a solution of 0.01% TAA as regular drinking water for the first 2 weeks. Afterwards, the dose was increased to 0.02% for 16 weeks. This fibrogenesis protocol was well tolerated by Foxn1 nu/nu mice and no mortality was observed in mice that received the full 16-week TAA protocol (n = 50).

**Fig 1:**
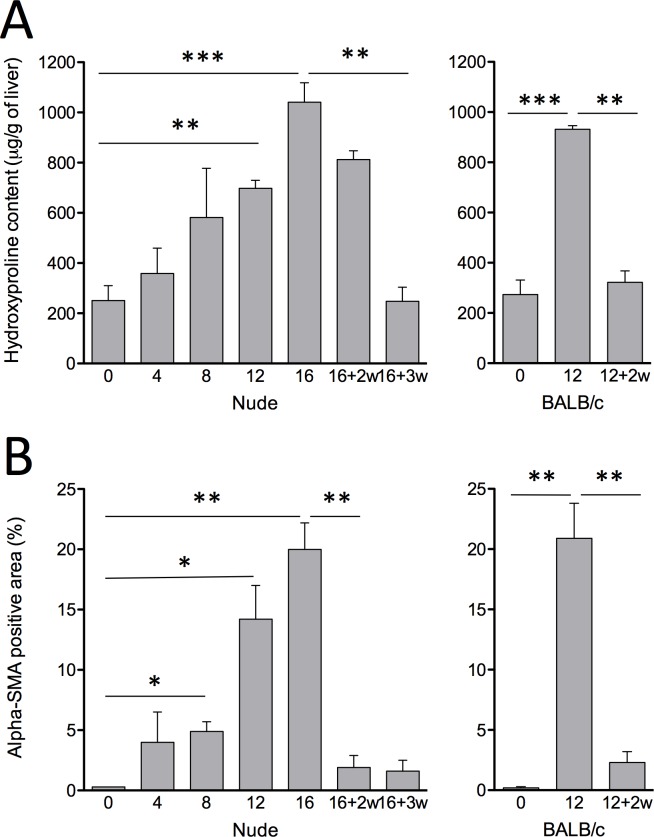
Liver fibrosis progression. (A) Hydroxyproline content of Foxn1 nu/nu mice (left) and BALB/c mice (right) liver samples following TAA fibrosis induction. (B) alpha-SMA image analysis representing percentage of positive pixels of total area from immunofluorescent stained liver slides images. Weeks of recovery are indicated as +nw. Results are expressed as mean ± SE. (*P<0.05, **P<0.01, ***P<0.001)

Fibrosis and collagen accumulation was assessed by hydroxyproline content overtime in this model. Collagen accumulation in Foxn1 nu/nu mice reach high levels 16 weeks after the beginning of 0.02% TAA administration (1041.2 ± 77.0μg/g of liver) (Fig 1). This level is similar to that achieved using the conventional 12 weeks IP protocol in BALB/c mice (931.6 ± 14.8μg/g of liver) (Fig 1). Regression of fibrosis occurred in Foxn1 nu/nu mice as evidenced by hydroxyproline levels reaching baseline 3 weeks after the end of TAA administration (Fig 1). HSC activation was evaluated by measurement of alpha-SMA positive areas (Fig 1B). Foxn1 nu/nu mice that had received oral TAA showed 20.0 ± 2.2% alpha-SMA positive areas after 16 weeks. This level is comparable to that of BALB/c mice that had received the IP treatment over a 12-week period (20.9 ± 2.9% alpha-SMA positive area) (Fig 1B). Alpha-SMA positive areas decreased significantly after cessation of TAA administration in Foxn1 nu/nu mice (1.9 ± 1.0%).

**Fig 2:**
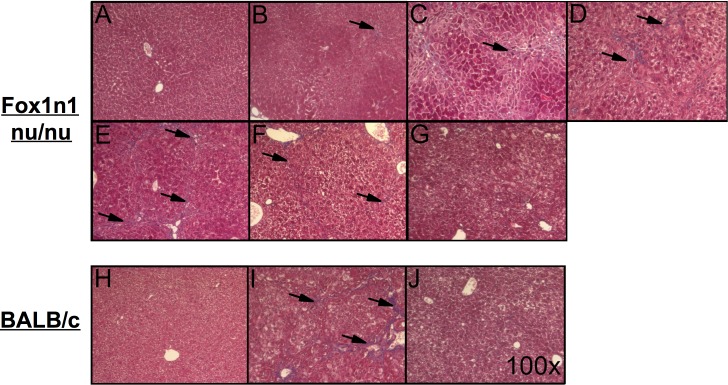
Histological features of liver fibrosis. Masson’s trichrome stained livers from Foxn1 nu/nu mice (top) receiving (A) normal drinking water, (B) TAA drinking water [0.02%] for 4 weeks, (C) 8 weeks, (D) 12 weeks, (E) 16 weeks, (F) 2 and (G) 3 weeks of recovery after the 16 week treatment and from BALB/c mice (bottom) IP injected with (H) saline, (I) TAA [200 μg/g bw] for 12 weeks and (J) 2 weeks of recovery following the 12 week TAA treatment. Pictures were obtained at a 100X magnification. Arrows indicate fibrotic septae.

Using Masson’s trichrome staining, evidence of progressive ECM accumulation could be observed during TAA administration in Foxn1 nu/nu mice (Fig 2B to 2E). Sixteen weeks of oral TAA administration (Fig 2E) in Foxn1 nu/nu mice led to similar levels of fibrosis to that of BALB/c mice that received 12 weeks of conventional IP TAA administration (Fig 2I). Histological signs of liver fibrosis decreased over a 3-week period (Fig 2F and 2G).

**Fig 3:**
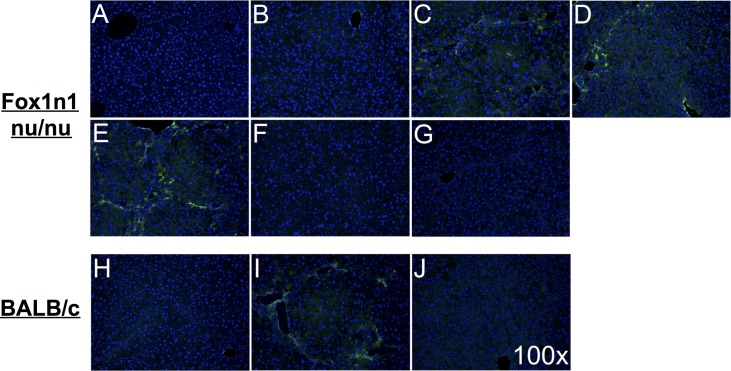
Activation of hepatic stellate cells. Histological alpha-SMA (green) immunofluorescent-stained livers from Foxn1 nu/nu mice (top) receiving (A) normal drinking water, (B) TAA drinking water [0.02%] for 4 weeks, (C) 8 weeks, (D) 12 weeks, (E) 16 weeks, (F) 16 weeks followed by 2 weeks or (G) 3 weeks of recovery and from BALB/c mice (bottom) IP injected with (H) saline or (I) TAA [200μg/g bw] for 12 weeks and (J) 2 weeks of recovery following TAA treatment. Nuclei were stained with Hoechst 33258 (blue). Microphotographs were obtained at a 100X magnification.

Activation of hepatic stellate cells was evaluated using alpha-SMA immunofluorescence staining (Fig 3). The proportion of alpha-SMA positive stellate cells progressively increased 4, 8, 12 and 16 weeks after initiation of TAA administration, along newly formed fibrous septae (Fig 3A to 3E). Following a 2-week recovery period, Foxn1 nu/nu showed absence of alpha-SMA positive cells (Fig 3F). The level of activated stellate cells and the time to recovery were similar to that of conventional BALB/c mice model.

**Fig 4:**
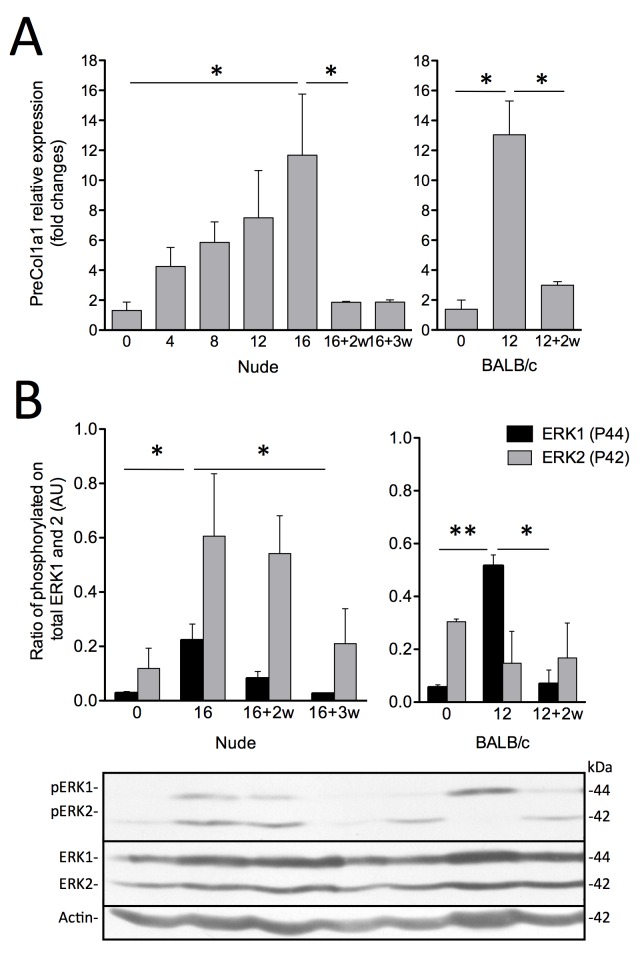
COL1 mRNA expression and ERK1/2 phosphorylation. (A) PreCol1a1 mRNA relative expression qPCR of Foxn1 nu/nu (left) and BALB/c mice (right) liver samples following induction of fibrosis and (B) ERK1/2 phosphorylation by Western blot of Foxn1 nu/nu (left) and BALB/c mice (right) liver samples following TAA fibrosis induction with associated representative pictures. Number of weeks of recovery is indicated as +nw. Results are expressed as mean ± SE. (*P<0.05, **P<0.01)

Fibrosis progression was also assessed by measuring preCol1a1 mRNA expression levels (Fig 4A). Levels increased progressively after the beginning of TAA administration and reached their highest level after 16 weeks (11.7 ± 4.1 fold changes; *P*<0.05 vs. control). These levels were comparable to the ones observed after 12 weeks in BALB/c mice (13.1 ± 2.3 fold changes; *P*<0.05 vs. control Foxn1 nu/nu mice). preCol1a1 levels returned to normal values 2 weeks after the end of TAA administration (1.9 ± 0.1 fold changes).

Next, activation of the MAPK pathway during liver fibrogenesis was evaluated by densitometric analysis of total and phosphorylated ERK1/2 levels (Fig 4B). No change was observed in total ERK1 or 2 levels, but phosphorylation of ERK1 was significantly increased after 16 weeks of TAA administration (0.22 ± 0.06 AU vs. control mice: 0.03 ± 0.01 AU; *P*<0.05) (Fig 4B). Phospho-ERK2 levels were also higher in fibrotic Foxn1 nu/nu mice than in control animals. After 3 weeks of recovery, phosphorylation levels of ERK1/2 returned to control levels in Foxn1 nu/nu mice (Fig 4B).

### Hepatoprotective effect of liver fibrosis to cytotoxic agents

**Fig 5:**
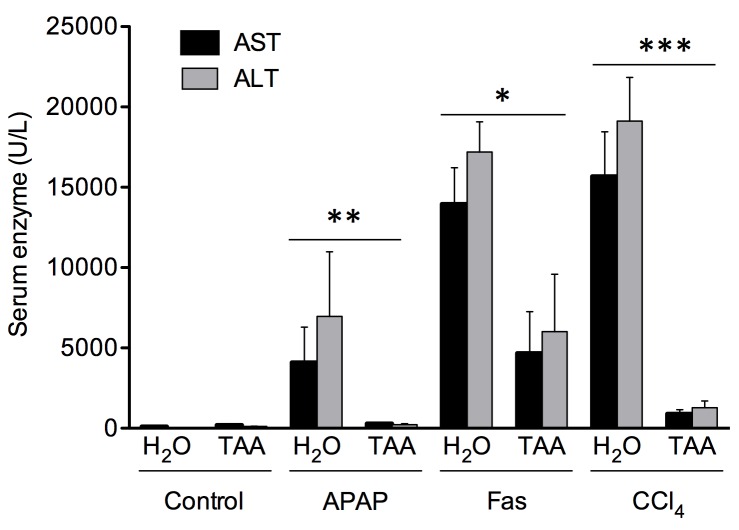
Protective effect of liver fibrosis in Foxn1 nu/nu mice against different cytotoxic agents. Dosage of serum AST and ALT levels from fibrotic Foxn1 nu/nu mice after TAA administration through drinking water [0.02%] for 16 weeks while non-fibrotic mice received normal drinking water (H_2_O). Cytotoxic agents were injected IP: CCl_4_ [0.5 mL/kg bw] (sacrificed 24 hours following injection), Fas Jo2 antibody [0.5μg/g bw] (sacrificed 6 hours following injection) and APAP [750 mg/kg bw] (sacrifice 6 hours after injection). Results are expressed as mean ± SE. (*P<0.05, **P<0.01, ***P<0.001)

In order to evaluate the sensitivity of fibrotic animals to hepatotoxic agents, ALT and AST serum levels were measured following administration of hepatotoxic compounds (Fig 5). AST levels were significantly lower in fibrotic (292 ± 66 U/L) versus non-fibrotic Foxn1 nu/nu mice (4087 ± 2205 U/L) 6 hours after APAP injection (*P*<0.01). Similar differences were observed 6 hours after agonist anti-Fas Jo2 antibody injection (4665 ± 2596 U/L in fibrotic animals vs. 13953 ± 2782 U/L in non-fibrotic animals; *P*<0.01) and 24 hours after CCl_4_ injection (fibrotic: 888 ± 268 U/L vs. non-fibrotic: 15673 U/L; *P*<0.001).

**Fig 6:**
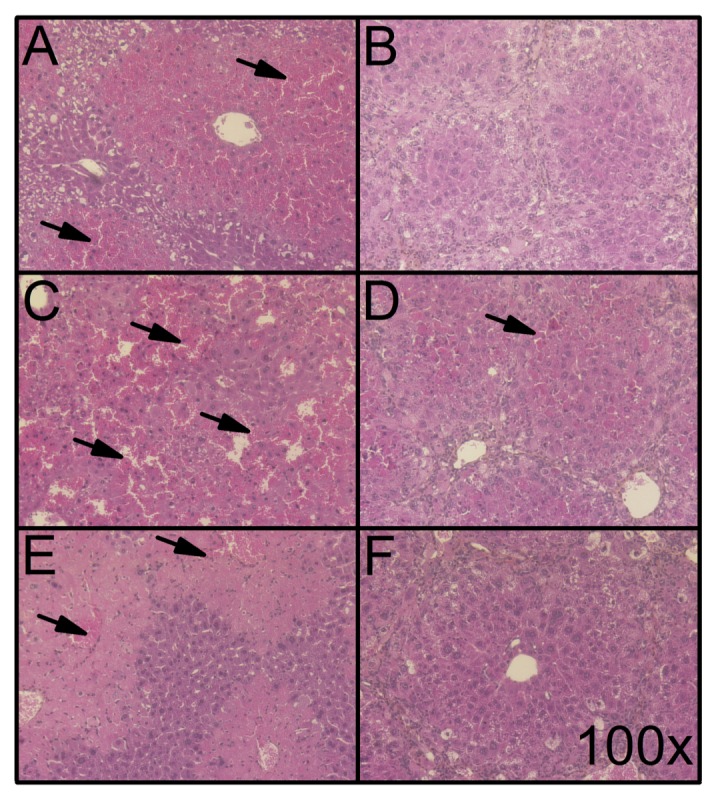
Histological features of hepatoprotective effect of liver fibrosis to cytotoxic agents. HPS-stained liver slices of Foxn1 nu/nu mice injected with the following cytotoxic agents: CCl_4_ [0.5 mL/kg bw] in (A) non-fibrotic and (B) fibrotic mice (16 weeks of TAA in drinking water [0.02%] (sacrificed 24 hours following TAA treatment); Fas Jo2 antibody [0.5 μg/g bw] in (C) non-fibrotic and (D) fibrotic mice (sacrificed 6 hours following TAA treatment); and APAP [750 mg/kg bw] in (E) non-fibrotic and (F) fibrotic mice (sacrificed 6 hours following TAA treatment). Representative microphotographs were obtained at a 100X magnification. Arrows indicate parenchymal red blood cell infiltration and/or necrosis.

Liver histology from animals that received hepatotoxic agents revealed that liver injury, such as red blood cell infiltration caused by APAP administration, was more apparent in non-fibrotic (Fig 6A) than fibrotic mice (Fig 6B). Histological signs of acute liver injury caused by anti-Fas Jo2 agonist antibody were also less important in fibrotic animals (Fig 6D) in comparison to control animals (Fig 6C). Finally, parenchymal necrosis was less apparent in fibrotic animals that were administered a single toxic dose of CCl_4_ (Fig 6F) compared to non-fibrotic animals (Fig 6E).

## Discussion

Administration of TAA through drinking water combined with step-wise dose increase can lead to a reproducible fibrogenic model in athymic mice without mortality. This is of prime importance in order to achieve adequate experimental observations in immunodeficient animals with liver fibrosis, for example in hepatocellular carcinoma models. Indeed, high mortality rate was consistently observed in a dimethylnitrosamine (DMN) model using NOD-SCID mice and also in a Foxn1 nu/nu mice model using IP injections of TAA [27, 33]. In the present study, using step-wise increased dosage of TAA and a more continuous delivery through drinking water, toxicity of TAA was reduced without undermining the extent of its fibrogenic impact. This delivery approach should be considered when working with immunodeficient or sensitive mouse strains.

Using this approach, Foxn1 nu/nu mice developed similar levels of fibrosis, according to Masson’s trichrome evaluation as well as hydroxyproline content measurements, as conventional immunocompetent models (IP-delivered TAA in BALB/c mice). Fibrosis developed over a 16-week period compared to 12 weeks using the conventional model in BALB/c mice. Recently, Hyon *et al*. showed that liver fibrosis in NOD-SCID mice (lacking both T and B cells) also needed a longer treatment period to reach adequate degrees of fibrosis [33]. Since both the delivery method and genetic background of the animals differed, we cannot conclude as to why Foxn1 nu/nu mice took longer to achieve similar level of fibrosis; however, the less aggressive TAA regimen is likely to influence the development of fibrosis.

Fibrogenesis in Foxn1 nu/nu mice was associated with increased levels of alpha-SMA along fibrous septa, a clear sign of HSC activation, the main producer of ECM in the liver [3, 34]. This activation is necessary for the production of ECM molecules such as COL1 [35]. In order to produce COL1, preCol1a1 mRNA levels are increased during fibrogenesis. PreCol1a1 levels were increased following a similar pattern and in concordance with hydroxyproline content measurements. The highest levels of transcription in Foxn1 nu/nu mice were reached after 16 weeks. T-cell impairment is generally not associated with altered fibrogenesis, but Th2 and Th17 CD4+ T cells can play a role in the inflammatory process leading to cytokine production and HSC activation [15, 36]. However, TGF-β1 remains the main determinant of HSC activation and stimulation of preCol1a1 mRNA production [10]. From our data, we can safely conclude that the absence of T cells in Foxn1 nu/nu mice is not associated with impaired HSC activation or fibrogenesis.

Fibrosis regression occurs in mice models when the fibrosis stimulus is removed. This process requires the deactivation and death of HSCs and a remodelling of ECM components to their normal ratios through specialised collagenases named matrix metalloproteinases (MMPs) [22, 23]. Two weeks after the end of TAA administration, alpha-SMA levels were similar to their respective non-fibrotic controls. Similarly, preCol1a1 levels returned to normal values two weeks after the end of TAA administration. However, Masson’s trichrome histology and hydroxyproline contents measurements showed that regression of fibrosis in Foxn1 nu/nu was complete 3 weeks after the end of treatment compared to 2 weeks for BALB/c mice with the conventional IP TAA protocol. HSC deactivation is an important part of fibrosis regression, but degradation of ECM is mainly due to restorative macrophage phagocytosis and MMPs activity, such as MMP-13 in mice [22, 37, 38], a process not known to be associated with T cells. The lack of T cell did not significantly alter or prevent fibrosis regression in these animals.

The increase in MAPK phosphorylation, especially the ERK1 subunit, has been shown to be a key feature of fibrosis and has been shown to be associated with the hepatoprotective state induced by liver fibrosis [25]. In Foxn1 nu/nu mice, phosphorylation of both ERK1 and 2 subunits increased. The protective effect of liver fibrosis to cytotoxic agents, associated with the activation of ERK1/2, was also similar to previously described models using immunocompetent mice [25]. From these data, we can safely conclude that the absence of mature T-cells does not play a significant role in the hepatocellular protective effect of liver fibrosis.

In conclusion, the induction of liver fibrosis in Foxn1 nu/nu mice by the addition of TAA in drinking water, in a step-wise dose increase, is a reproducible model of liver fibrosis in immunodeficient animals. This model also reproduces the MAPK-mediated hepatoprotective effects described in immunocompetent mice and thus offers a promising model for the evaluation of the impact of liver fibrosis on pathologies such as hepatocellular carcinoma.
